# Caryophyllene Oxide, a Bicyclic Terpenoid Isolated from *Annona macroprophyllata* with Antitumor Activity: In Vivo, In Vitro, and In Silico Studies

**DOI:** 10.3390/ijms252413355

**Published:** 2024-12-12

**Authors:** Jesica Ramírez-Santos, Fernando Calzada, Normand García-Hernández, Elizabeth Barbosa, Claudia Velázquez, Miguel Valdes

**Affiliations:** 1 Unidad de Investigación Médica en Farmacología, UMAE Hospital de Especialidades, Centro Médico Nacional Siglo XXI, Instituto Mexicano del Seguro Social, Mexico City CP 06720, Mexico; valdesguevaramiguel@gmail.com; 2 Unidad de Investigación Médica en Genética Humana, UMAE Hospital Pediatría, Centro Médico Nacional Siglo XXI, Instituto Mexicano del Seguro Social, Mexico City CP 06725, Mexico; normandgarcia@gmail.com; 3 Escuela Superior de Medicina, Instituto Politécnico Nacional, Mexico City CP 11340, Mexico; rebc78@yahoo.com.mx; 4 Área Académica de Farmacia, Instituto de Ciencias de la Salud, Universidad Autonoma del Estado de Hidalgo, San Agustin Tlaxiaca CP 42076, Mexico; cvg09@yahoo.com; 5 Laboratorio de Biofísica y Biocatálisis, Sección de Estudios de Posgrado e Investigación, Escuela Superior de Medicina, Instituto Politécnico Nacional, Mexico City CP 11340, Mexico

**Keywords:** caryophyllene oxide, antilymphoma activity, U-937 cells, acute oral toxicity, molecular docking, *Annona macroprophyllata*

## Abstract

The Annona genus contains some species used in Mexican traditional medicine for the treatment cancer, including *Annona macroprophyllata (A. macroprophyllata)*. The present study aimed to investigate the anticancer activity of caryophyllene oxide (CO) isolated from *A. macroprophyllata* using in vivo, in vitro, and in silico approaches. The identification of CO was performed using gas chromatography-mass spectroscopy and NMR methods. Antilymphoma activity was evaluated in male and female Balb/c mice inoculated with U-937 cells. Cytotoxic activity was evaluated using the WST method and flow cytometry was used to determine the type of cell death. Acute oral toxicity was determined, and a molecular docking study was performed using target proteins associated with cancer, including, HMG-CoA, Bcl-2, Mcl-1, and VEGFR-2. Results showed that CO exhibited significant antilymphoma and cytotoxic activities, and its effects were comparable to MTX. In addition, flow cytometry showed that the anticancer activity of CO could be mediated by the induction of late apoptosis and necrosis. The result for the acute oral toxicity of CO was classified in category 4, suggesting it is low risk. Finally, molecular coupling studies showed that CO had more affinity for the enzymes HMG-CoA reductase and Bcl-2. Our study provides evidences that CO is a potential anticancer agent for the treatment of histiocytic lymphoma.

## 1. Introduction

Medicinal plants are the source of at least 60% of the drugs currently used for the treatment of cancer. Some of the active molecules isolated from plants are alkaloids, diterpenes, and lignans [1]. From 1981 to 2019, 247 cancer drugs were approved worldwide, however, 64.9% were obtained from natural products. These were classified as follows; 62 natural products, 43 are derived from a natural product and is usually a synthetic modification, 13 were made by total synthesis, but the pharmacophore is/was of a natural product and 45 are synthetic/imitation of a natural product drug [2]. In Mexico, 90% of the population has opted for one of these 4500 medicinal plants at least once in their lives. However, only 5% of these plants have been pharmacologically analyzed [3]. These data indicate that it is crucial to continue carrying out experimental tests that allow us to know the chemical elucidation of their active ingredients, pharmacological activity, toxicological studies, pharmacokinetic properties, and mode of action.

**Figure 1:**
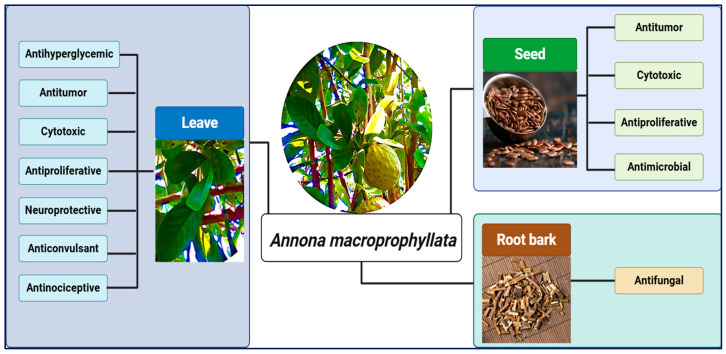
Pharmacological activities reported for the leaves, seeds and root bark of *Annona macroprophyllata*.

*Annona macroprophyllata* Donn (*A. macroprophyllata*) belongs to the Annonaceae family, and it is used in traditional medicine to treat diabetes, inflammation, pain, and cancer [1,4]. Some pharmacological studies have reported that *A. macroprophyllata* has antihyperglycemic, anticonvulsant, antifungal, antinociceptive, antimicrobial, and antitumor activity (Figure 1) [4,5,6,7,8]. The metabolites responsible for its pharmacological properties correspond to natural products such as alkaloids, flavonoids and terpenoids [8,9,10,11,12,13,14]. As for the cytotoxic and antitumor activity of *A. macroprophyllata* on the cell lines SW-480 (colon adenocarcinoma), HeLa (cervical carcinoma), and chemical-induced colorectal cancer, it has been attributed to acetogenins such as laherradurin and kerimoline-2 [6,14]. In addition, the antitumor and cytotoxic activity of *A. macroprophyllata* were demonstrated in vivo and in vitro approaches using an NHL mice model and on U-937 cells (histiocytic lymphoma), where the terpenoids, geranylgeraniol, phytol, and farnesyl acetate were responsible for these effects [15,16].

In the last decade, the pharmacological properties of some terpenoids have been studied, as scientific studies have reported that they possess antiproliferative and cytotoxic activity in several cancer cell lines, and that their anticancer effects act on the initiation and progression of cancer [17]. Including cell proliferation, cell differentiation, cell death, angiogenesis, and metastasis [18,19]. Also, among the mechanisms of action that have been described are cell cycle arrest in the G0/G1 phase and mitochondrial depolarization; apoptosis mediated by the activation of caspases of the mitochondrial pathway; reduction of topoisomerase I and II followed by DNA damage; downregulation of Bcl-2 and alteration of the p53 gene; inhibition of signaling pathways involved in cell survival, such as MAPK and PI3K/Akt, and inhibition of the NF-K B pathway [18,19,20].

Non-Hodgkin’s lymphoma (NHL) includes about 60 types of solid hematological cancers that affect organs of the lymphatic system [21]. According to the Global Cancer Observatory (GLOBOCAN), the mortality statistics for NHL in 2020 were 259,793, and the number of new cases was 573,278 worldwide, with a higher incidence and mortality in men than in women [22].

It is well known that, in most lymphomas, overexpression of the biomarkers Bcl-2 and Mcl-1 is promoted [21]. These biomarkers belong to the BCL-2 family, and they have a role in the regulation of cell survival and death. However, in some types of cancer they become oncogenes that prevent cell death by apoptosis. Given this context, the interest in these targets has grown in recent years [22]. In addition, it has been proposed that targeting these biomarkers could have an impact on chemotherapy resistance in several types of lymphomas [21,23,24]. Another target in cancer is the overexpression of the vascular endothelial growth factor 2 receptor (VEGFR-2), as this is the protein to which VEGF binds for activation. This binding promotes angiogenesis; nonetheless, the development of new blood vessels is an essential part of tumor cell growth and metastasis [25]. Additionally, other research has correlated the expression of VEGFR-2 and Bcl-2, as studies have shown that VEGFR-2 is responsible for improving endothelial cell survival through the regulation of Bcl-2 expression through the phosphatidylinositol 3-kinase/Akt signaling pathway [26]. Moreover, it has been proposed that the enzyme HMG-CoA reductase could be a target in some types of cancer [27]. By analyzing statin activity in preclinical experiments with T-lymphomas, prostate cancer, and pancreatic cancer, they reportedly observed an improvement in tumor inhibition. Studies propose that inhibition of the enzyme HMG-CoA reductase inhibits cholesterol synthesis and, consequently, the reduction of tumor androgen signaling; this results in a decrease in lipid rafts in tumor cells and the lack of prenylation of G, Ras, and Rho proteins by reducing the synthesis of intermediates such as geranyl pyrophosphate and farnesyl pyrophosphate [27,28,29].

In a previous work, we evaluated the anti-lymphoma effect of petroleum ether extract of the leaves from *A. macroprophyllata,* and of geranylgeraniol, phytol, and farnesyl acetate, three of its isolated acyclic terpenoids. Continuing the objective of obtaining other antitumor agents that support the antitumor activity of *A. macroprophyllata*, in this work the sesquiterpene caryophyllene oxide (CO) was isolated and identified. In addition, we seek in this study to explore its antilymphoma activity via in vitro and in vivo models including its cytotoxic properties against the U-937 cell line and its induction of apoptosis. Also, we explored its margin of safety through acute oral toxicity testing, and toxicological and pharmacokinetic evaluations using computer tools. Finally, molecular docking studies were performed using cancer-associated proteins as targets, including HMG-coA reductase, Bcl-2, Mcl-1, and VEGFR-2.

## 2. Results

### 2.1. Analysis of GC-MS and ^1^H-^13^C-NMR Spectra of Caryophyllene Oxide

**Figure 2:**
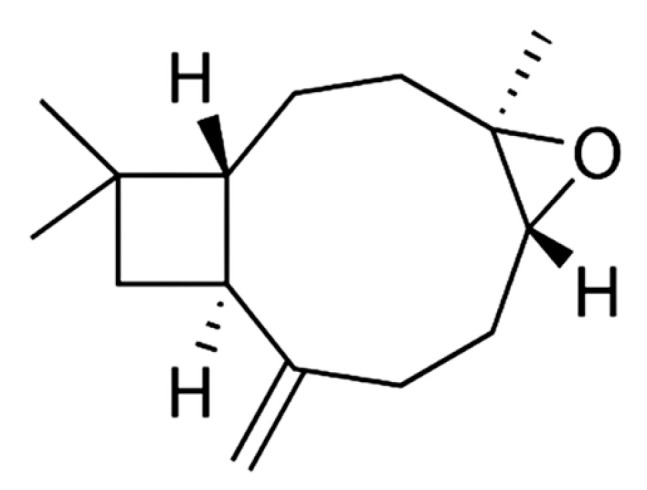
Structure of caryophyllene oxide obtained from petroleum ether extract from *A. macroprophyllata* leaves.

**Figure 3:**
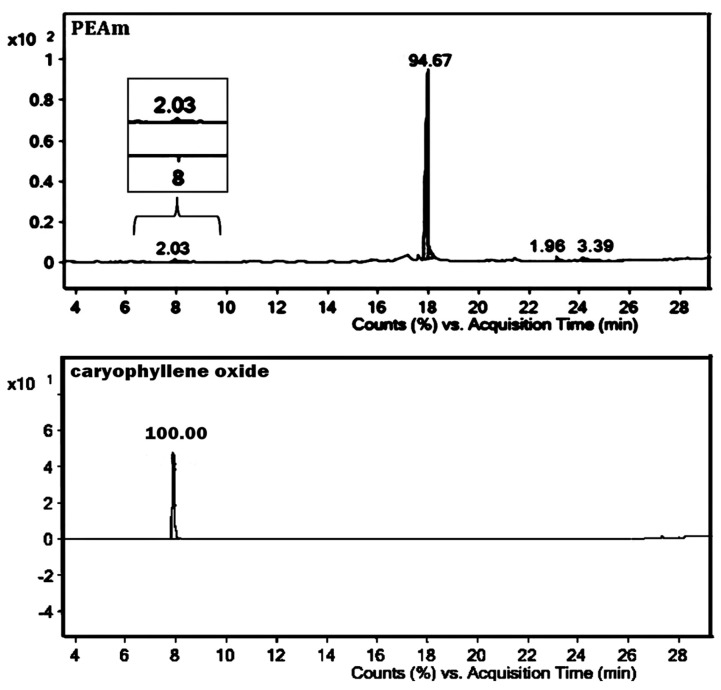
Gas chromatography-mass spectrometry analysis of petroleum ether extract from *A. macroprophyllata* leaves (PEAm) and caryophyllene oxide standard. The x-axis indicates the retention time in minutes, while the y-axis indicates the peak % signal intensity.

**Figure 4:**
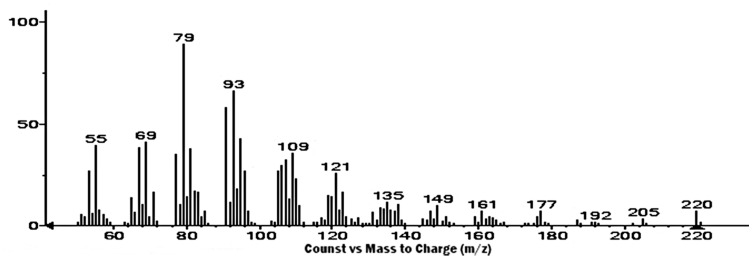
Mass spectrum of caryophyllene oxide.

**Figure 5:**
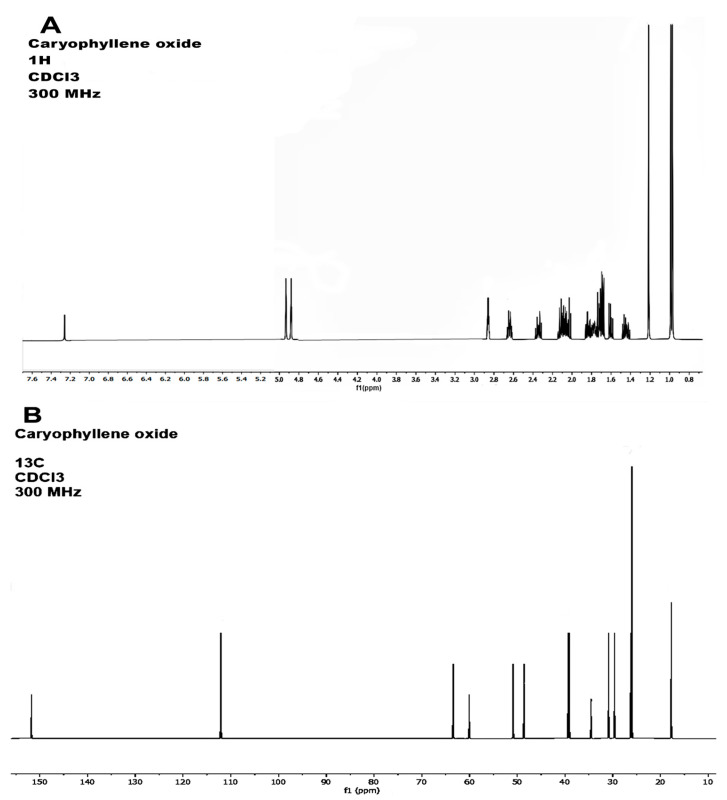
^1^H-NMR spectra (**A**) and ^13^C-NMR spectra (**B**) of caryophyllene oxide.

Petroleum ether extract from *A. macroprophyllata* (PEAm) leaves was analyzed by GC-MS, and an authentic sample of caryophyllene oxide from Sigma was used as standard (Figure 2). The results showed the presence of the bicyclic sesquiterpene caryophyllene oxide (CO) at 7.93 min (Figure 3) (Table 1). Identification was obtained by comparing their mass spectra with the mass spectra of the NIST library database. The mass spectrum showed a characteristic fragmentation pattern for the sesquiterpene (Figure 4), fragments at *m*/*z* 79.05, *m*/*z* 93.05 and *m*/*z* 107.05, obtaining a peak of molecular ions at *m*/*z* 220.05. The *m*/*z* ion 109 (diagnostic ion) is the *m*/*z* ion 93 but with an additional oxygen molecule, which infers the structure of a sesquiterpene. The ion that confirmed the fragmentation of sesquiterpene-like molecules was the *m*/*z* ion 93.05 derived from the formation of the *m*/*z* ion 136 and the loss of a propene radical (*m*/*z* 43). The base peak ion *m*/*z* 79 is formed by the loss of a methylene group of the *m*/*z* 93 ions. The results were similar to those obtained in the literature [30,31]. Subsequently, the identification of CO was confirmed by 1 H and ^13^C NMR spectroscopic technique (Figure 5). The ^13^C-NMR spectrum showed signals ranging δ 17.75–112.07 ppm, yielding that the molecule contains 15 carbons including the three methyl groups and a methylene group that belongs to an exocyclic double bond. The carbon of the exocyclic double bond appeared in the lowest region at 112.07 δ ppm, carbon 14 and 15 (adjacent methyl) appeared in the highest region 26.0 and 26.1 δ ppm, respectively. On the other hand, the ^1^H-NMR spectrum showed signals from 0.97 to 4.93 ppm. Similarly, it indicated a most downfield multiplet signal at 4.93 δ ppm for the adjacent methyl protons indicated at position 12. The methylene group indicated by 13 gave a single-type signal at 1.21 δ ppm. The protons of the adjacent methyls indicated at position 14 and 15 appeared in the high region of the field at about 0.97 to 0.99 δ ppm (Table 2). Finally, all the signals (NMR ^1^H and ^13^C^)^ of the isolated compound were compared with those reported in the literature [32,33] and were confirmed by MNova from MestreLab (version 14.0). In addition, when a presumptive identification was made by thin layer chromatography, the same retention factor was obtained when comparing the isolated PEAm compound with the true sigma compound. 

### 2.2. Cytotoxic Activity

**Figure 6:**
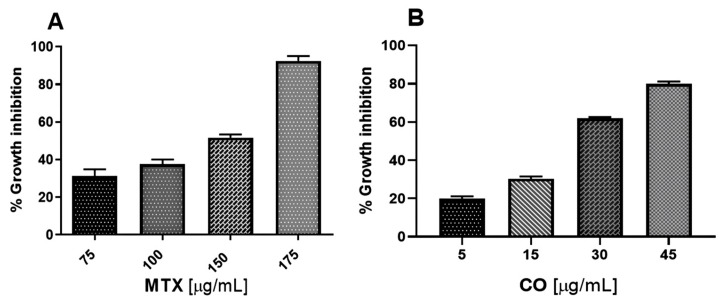
Cytotoxic activity of CO in the U-937 cell line. The graphs show the inhibition of cell growth caused by methotrexate (**A**) and caryophyllene oxide (**B**) at different concentrations after 24 h of exposure. The assays were performed in triplicate and these data were used to calculate the CC_50_ by lineal regression analysis.

The results for cytotoxic activity against the U-937 cell line showed that CO and MTX showed dose-dependent cytotoxic activity (Figure 6). 

In addition, CO (CC_50_ 24.25 ± 0.37 μg/mL) obtained a better cytotoxic activity compared to MTX (CC_50_ 118.87 ± 0.26 μg/mL) (Table 3).

### 2.3. Antilymphoma Activity

**Figure 7:**
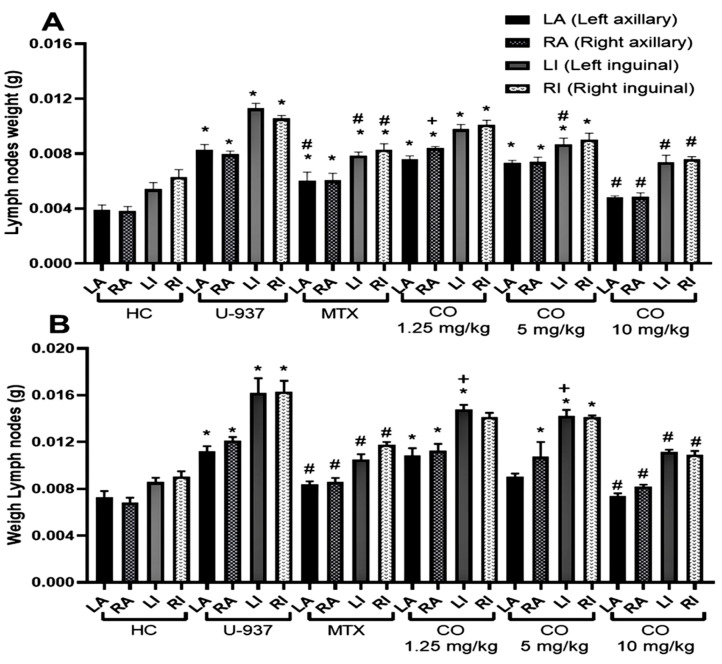
Lymph node weights (g) of female mice (**A**) and male mice (**B**). Healthy control (HC tween 80, 2% *v*/*v* in water), untreated control (U-937), methotrexate (MTX) 1.25 mg/kg and caryophyllene oxide (CO) at 1.25, 5 and 10 mg/kg. The graph shows the weight of the axillary and inguinal lymph nodes as follows: left axillary (LA), right axillary (RA), left inguinal (LI), and right inguinal (IR). Results obtained by ANOVA one-way analysis followed by Dunnett’s test for multiple comparison. Data are expressed as mean ± SEM, (*n* = 6); * *p* < 0.05 vs. HC, # *p* < 0.05 vs. U-937, + *p* < 0.05 vs. MTX.

To evaluate the antilymphoma activity of CO and MTX, the weight of the axillary and inguinal lymph nodes of female and male mice with NHL were obtained (Figure 7), and the percentages of inhibition of lymph node growth after treatment administration were calculated. CO showed significant antilymphoma activity at a dose of 10 mg/kg in both sexes, the results were similar in female and male mice. The growth inhibition of axillary and inguinal lymph nodes was 72.31 ± 1.77% and 75.55 ± 1%, respectively. For MTX, the highest percentage of growth inhibition of axillary and inguinal lymph nodes was obtained at a dose of 1.25 mg/kg; in female and male mice, the results were 53 ± 4.54% and 68 ± 1.09%, respectively. 

When calculating the ED_50,_ we found that MTX obtained an ED_50_ five times lower than CO for both sexes (Table 4). The groups of female mice treated with MTX and CO obtained an ED_50_ of 1.24 ± 0.038 mg/kg and ED_50_ of 6.14 ± 0.52 mg/kg, respectively. In the case of male mice, the results were ED_50_ 0.94 ± 0.023 mg/kg and ED_50_ 5.46 ± 0.42 mg/kg, respectively. Overall, we found that CO and MTX produced dose-dependent antilymphoma activity in both sexes.

### 2.4. Acute Oral Toxicity

Acute oral toxicity results revealed that CO was classified as category 4, obtaining an LD_50_ > 1000 mg/kg and therefore low risk. When monitoring the animals after intragastric CO administration, the animals presented lethargy during the first 30 min. Subsequently, the animals showed no signs of toxicity, and there was no mortality in the animals. When performing the macroscopic analysis, no tissue lesions were observed, and when comparing the weights of the organs (stomach, intestine, spleen, liver and kidneys), no significant differences in gain or decrease were observed compared to the control group. In addition, the results of acute oral toxicity for MTX indicated that it corresponds to category 4 with an LD_50_ of 335.04 ± 0.39 mg/kg. In the macroscopic study, no signs of toxicity were found in the removed organs; however, it did cause mortality in the animals during the study. With the results obtained, the TI (therapeutic index) for CO and MTX was calculated. The TI values for CO were close for both sexes and were >150, suggesting that it has a wide margin of safety for use. In the case of MTX, IT outcomes were higher compared to CO (Table 5).

### 2.5. Induction of Apoptosis

**Figure 8:**
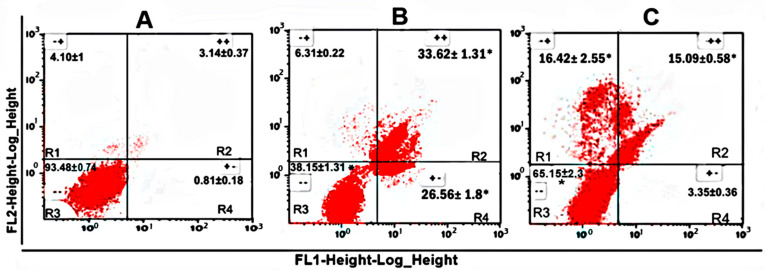
Apoptosis assay using annexin V/PI staining. The apoptotic and necrotic effect of CO is observed. U-937 cells were exposed for 24 h to, vehicle 0.02% dimethyl sulfoxide (DMSO) (**A**), methotrexate (CC_50_ 118.87 µg/mL) (**B**), and caryophyllene oxide (CC_50_ 24.25 µg/mL) (**C**). The histogram zones indicate the following: R1 = necrosis, annexin V-FITC negative/PI positive (− +); R2 = late apoptosis, annexin V-FITC positive/PI positive (+ +); R3 = viable cells, annexin V-FITC negative/PI negative (− −); R4 = early apoptosis, annexin V-FITC positive/PI negative (+ −). The experiments were conducted in triplicate. Data are expressed as means ± SEM, *n* = 3; *p < 0.05 vs DMSO.

Once the antilymphoma activity of CO had been determined, we decided to evaluate whether its antitumor effect could be mediated by the induction of apoptosis (Figure 8). The analysis for the detection of apoptosis by flow cytometry showed that CO and MTX generated an increase in the percentage of cell death in late apoptosis (R2 quadrant), obtaining values of 15.09 ± 1.26% and 33.62 ± 1.31% compared to DMSO (untreated control). In the case of early apoptosis (R4 quadrant), CO showed a weak effect on U-937 cells; in contrast, MTX produced an increase in the percentage of cells in early apoptosis, obtaining a value of 26.56 ± 1.8% compared to DMSO. Finally, for necrotic cells (R1 quadrant), the results showed that CO generated an increase in the percentage of necrotic cells or cells that were not positive for annexin V staining and therefore did not die from apoptosis. In contrast, MTX did not generate a significant increase in the percentage of necrotic cells, obtaining a value of 6.31 ± 0.22%. The results indicate that CO induced late apoptosis and necrosis in U-937 cells. In the case of MTX, the data obtained showed that it induced early and late apoptosis in U-937 cells.

### 2.6. Toxicoinformatic and Pharmaceutical Analysis of Caryophyllene Oxide

After analyzing the results of the in vivo and in vitro CO experiments, the predictive values of some physicochemical, pharmacokinetic and toxicological properties were obtained using computer tools. The predictive values obtained showed that CO respects all the pharmacological similarity rules of Lipinski, Ghose, Veber, and Egan. In general, the pharmacokinetic parameters showed optimal and accepted levels, human intestinal absorption obtained a high value, it is poorly permeable to the blood–brain barrier, the value of its volume of distribution was 1.27 L/kg and the binding to plasma proteins was 82.26%. Metabolism values indicated that CO is an inhibitor of CYP2C19 and CYP2C9, and a substrate of CYP2C19 and CYP2D6. For half-life and clearance, the values obtained were acceptable. On the other hand, the toxicological information provided suggests that CO is not mutagenic and carcinogenic, as it does not cause hepatotoxicity in humans and is classified as class V, that is, it may be harmful if swallowed (Table 6).

### 2.7. Molecular Docking Studies of Caryophyllene Oxide

We performed molecular docking studies with the aim of understanding the anti-lymphoma properties of CO. Potential targets associated with cancer were HMG-CoA reductase, Bcl-2, Mcl-1, and VEGFR-2. The analysis of HMG-CoA reductase was considered, because it has a regulatory role in the oncogenic process by providing intermediates that serve in the post-translational modification of several proteins involved in aberrant transformation in several types of cancer [27,34]. Bcl-2 and Mcl-1 are proteins that have an important role in the regulation of cell survival and death; however, it has been reported that in NHL, they are overexpressed, triggering the evasion of apoptosis and chemoresistance [35,36]. Another target used was VEGFR-2, an enzyme that plays a role in angiogenesis and is therefore important in the growth of cancer [37].

**Figure 9:**
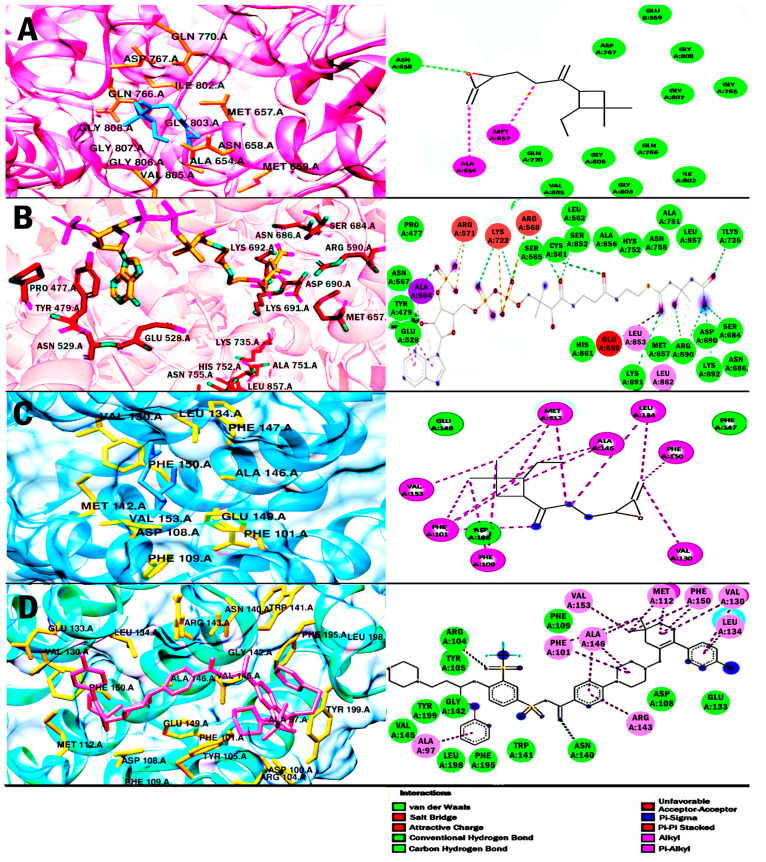
Results of molecular docking. The images show the binding site position and the 3D, and 2D interactions for caryophyllene oxide on each target. Caryophyllene oxide (blue) and interactions with amino acid residues (orange) in HMG-CoA reductase (**A**); and HMG-CoA substrate (yellow) and interactions with amino acid residues (red) in HMG-CoA reductase (**B**). For Bcl-2 protein, caryophyllene oxide (blue) (**C**); and navitoclax (pink) (**D**), in both cases interactions with amino acid residues (yellow) are highlighted. 2D orange-red interactions are unfavorable interactions, green interactions are polar-type interactions, and lilac interactions are nonpolar interactions.

**Figure 10:**
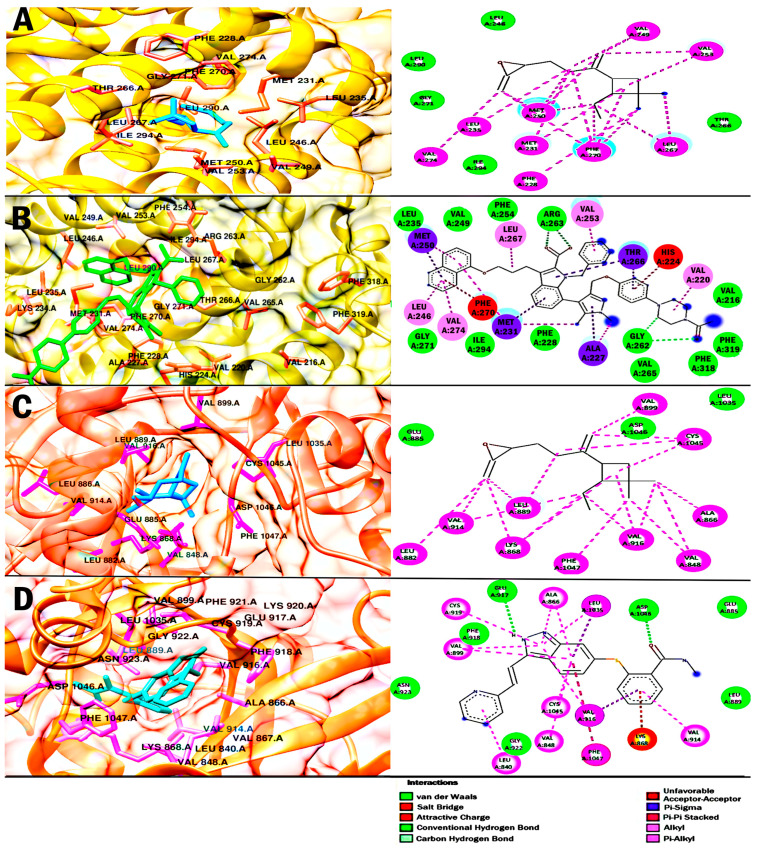
Results of molecular docking. The images show the binding site position and the 3D, and 2D interactions for caryophyllene oxide on each target. For Mcl-1, caryophyllene oxide (blue) (**A**); and 9EAs (green)(**B**), in both cases interactions with amino acid residues (orange) are highlighted. For VEGFR-2, caryophyllene oxide (blue) (**C**); and axitinib (green) (**D**), in both cases interactions with amino acid residues (lilac) are highlighted. 2D orange-red interactions are unfavorable interactions, green interactions are polar-type interactions, and lilac interactions are nonpolar interactions.

The results of the molecular docking analysis are shown in Table 7 (Figure 9 and Figure 10). The binding energy of CO with the enzyme HMG-CoA reductase was −7.85 kcal/mol. Some of the most important polar-type interactions were Asn 658, Asp 767, Gln 770, Ile 802, and Gly 808, and the non-polar interaction was in the Met 657 residue. To demonstrate that the binding of CO with HMG-CoA reductase was sufficient for its possible inhibition, we performed a comparison with the HMG-CoA substrate. The interaction of the HMG-CoA substrate with HMG-CoA reductase was −9.21 kcal/mol. Although CO only shared the Met 657 residue with the HMG-CoA substrate, it is inferred that CO could act as an inhibitor, as it bound within the catalytic portion of the HMG-CoA reductase enzyme (426–888 residues).

In addition, the results of the binding energy for CO with proteins of the BCL-2 family were shown to be close. For Bcl-2 and Mcl-1, a favorable binding energy of −7.47 kcal/mol and −6.77 kcal/mol, respectively, was obtained. For Bcl-2, the inhibitor navitoclax was used for molecular docking validation and comparison of its interactions with CO. The binding energy for navitoclax was −12.54 kcal/mol. In addition, the analysis showed that CO and navitoclax shared a polar-like interaction in the Asp 108 residue and several non-polar-like interactions in Phe 101, Phe 109, Met 112, and Ala 146, important residues of the hydrophobic groove of Bcl-2. For Mcl-1, the binding energy obtained by the inhibitor 9EA was higher than that obtained by CO with a value of −10.77 kcal/mol. However, CO obtained a greater number of nonpolar interactions than it shared with 9EA. The residues shared were Met 231, Val 249, Met 250, Val 253, Leu 267, Phe 270, and Val 274; these residues are part of the binding pocket for Mcl-1.The data obtained could suggest that CO allosterically inhibits Bcl-2 and Mcl-1 and counteract their antiapoptotics activities.

Finally, the binding energy of CO with VEGFR-2 was −5.8 kcal/mol, while the inhibitor axitinib obtained a more favorable binding with a value of −8.57 kcal/mol. However, CO shared some interactions with axitinib, these interactions were polar in the Glu 885 and Asp 1046 residues, and there were several non-polar interactions in the Val 848, Ala 866, Lys 868, Val 899, Val 914, Val 916, Cys 1045, and Phe 1047 residues. It should be noted that the Cys 1045 residue gives a DFG-out conformation to VEGFR-2, so it is inactive. In addition, CO has a fraction located in the hydrophobic back pocket, so we suggest that CO could stabilize the DFG-out conformation, and keep VEGFR-2 inactive.

## 3. Discussion

In the last decade, NHL has had an increase in incidence and mortality rate [22]. Lymphomas are classified as indolent and aggressive and are currently potentially curable with the use of chemotherapy or chemoimmunotherapy. However, treatment regimens are not effective in some subtypes of aggressive lymphomas, and in some cases, patients relapse and develop into refractory lymphoma as a result [22,38]. For this reason, the search for antitumor agents that have greater efficacy and safety remains a challenge.

In this work, the antilymphoma activity of the sesquiterpene caryophyllene oxide (CO) was evaluated using in vivo, in vitro, and in silico approaches. Antitumor activity was evaluated using a 65-day induced model and its cytotoxic and death-like properties by flow cytometry. We also evaluated their toxicity effects in an in vivo model and using computer tools. To obtain findings on their possible targets, we performed a molecular docking study using proteins related to cancers such as NHL.

First, isolation and purification of the sesquiterpene CO was performed by preparative thin layer chromatography from the extract of the leaves of *Annona macroprophyllata* (PEAm). It should be noted that the antilymphoma activity of PEAm was evaluated in our previous study [16]. The characterization focused on obtaining volatile compounds of a terpene nature due to the history of *A. macroprophyllata*, since in our previous study, acyclic terpenoids were obtained and showed antitumor activity [15,16]. The retention factor obtained for caryophyllene oxide was 7.93 min (Figure 3) and was the same for the authentic sample (Sigma-Aldrich). In addition, we structurally characterized CO using spectroscopic NMR methods (Table 2). We identified the product as the sesquiterpene CO (Figure 4). To our knowledge, CO has not been previously identified in *A. macroprophyllata*. However, in other species of the genus Annona, the identification of CO in low concentrations has been reported in *A. muricata* [39], *A. glabra* [40], and *A. squamosa* [41,42].

Once the identification of CO was made, its cytotoxic activity against U-937 cells was evaluated. This cell line is classified as an NHL and corresponds to a histiocytic lymphoma [43]. The results showed that CO was more cytotoxic compared to the reference drug methotrexate (MTX), as MTX obtained more than five times as much CC_50_ compared to CO (Table 3 and Figure 6). The cytotoxic activity of CO is consistent with other studies, as CO is reported to be cytotoxic in several cell lines such as human cervical adenocarcinoma (HeLa), human leukemia (HepG2), human lung cancer (AGS), human gastric cancer (SNU-1), and human stomach cancer (SNU-169), obtaining a CC_50_ of 3 to 27 μM [44]. Other studies that support our results are the antiproliferative activity of *A. macroprophyllata* in colon adenocarcinoma (SW-480) and cervical carcinoma (HeLa) [6]. Additionally, the results obtained from other species of the genus Annona, including the cytotoxic activity of *A. reticulata* against adult T-cell leukemia/lymphoma [44], *A. muricata* and *A. cherimolla* against the Ramos-1 lymphoma cell line, and Dalton lymphoma [45,46], and *A. squamosa* against adult T-cell leukemia/lymphoma, and K-562 leukemia cells [45,47]. All these findings report that *A. macroprophyllata* and CO have a broad spectrum of cytotoxicity. However, one of the limitations we consider in cell culture trials is the lack of stromal components.

Subsequently, antilymphoma activity was evaluated by comparing the effect of CO and the reference drug MTX selected as a commonly used treatment in Mexico to treat some NHL [48]. The results revealed that MTX was more potent than CO and that its activity was dose-dependent in both sexes (Table 4 and Figure 7). The increase in lymph node growth was observed to be significant for all four nodes in the U-937 group compared to the healthy control. In addition, OC obtained a significant difference in the decrease in nodal weight at the dose of 10 mg/kg in both sexes compared to U-937. There is little reported evidence of the in vivo antitumor effect of CO. However, one study supports our findings, as CO was shown to have a dose-dependent effect on HCCLM3 cell-induced tumors (adult hepatocellular carcinoma) [49]. Nevertheless, there are several studies demonstrating the anticancer activity of CO in some cancer cell lines, such as human lung cancer (A549) [50], human prostate cancer (PC-3) [51], and breast cancer (MCF-7) [52]. It is important to point out some limitations of our in vivo model; the interactions of the human tumor microenvironment are not well established, the variability in the in vivo response is due to the biological complexity of tumor models, and the rate of cell proliferation.

On the other hand, the assessment of acute oral toxicity showed that CO and MTX were classified as category 4 according to OECD guideline 423 (Table 5) [53]. After CO administration, lethargy was observed in the first 30 min in some animals, which could have been due to their high lipophilicity and the crossing of the blood–brain barrier. However, it did not cause mortality in the animals. Gross necropsy of the organs (stomach, intestine, liver, spleen, and kidneys) showed no evidence of any signs of toxicity, such as injuries, inflammation, necrosis, weight loss or gain compared to the control group. Acute oral toxicity testing is widely recommended when knowledge of DL_50_ and DL_1_ is required. However, one of the limitations of our study is that the administration of the compound to be evaluated is administered only once in the animals and the results could change when another study is carried out at repeated doses.

On the other hand, to support what was found in the acute oral toxicity test, computerized predictions of pharmacokinetic and toxicological properties were obtained (Table 6). The results were consistent with what was observed, CO has no mutagenic, carcinogenic, or hepatotoxic effects in humans. In addition, CO obtained an adequate pharmacokinetic profile by not violating any Lipinski rules [54]. Regarding the results for MTX, no signs of toxicity were observed during the 4 h of observation after administration; however, it did generate mortality in the animals in the following days. The TI values for CO were close for both sexes and were >150, suggesting that it has a wide margin of safety for use in humans. In the case of MTX, IT was higher compared to CO. However, the advantage of CO was observed in our repeated dose study when evaluating its antilymphoma activity, as it did not generate mortality in the animals. In contrast, MTX did generate mortality in some treated animals, which coincides with its serious side effects, such as mucosal ulceration, increased risk of infection, fatigue, fever, leukopenia, gastrointestinal bleeding, pancreatitis, cirrhosis, aplastic anemia, and malignancies [55].

Subsequently, we decided to evaluate whether its antitumor effect could be mediated by the induction of apoptosis by flow cytometry using staining with annexin V-FITC and propidium iodide (PI) as fluorescent dye (Figure 8). At 24 h of exposure, MTX generated a greater significant increase in early apoptosis and late apoptosis. While CO generated an increase in the percentage of U-937 cells in late apoptosis and necrosis. We believe that the increase in the cell population in early apoptosis was not observed significantly due to the time of exposure of the sesquiterpene. For the generation of necrosis, we believe it is due to the concentration and cell type. Some reports report that CO generated a significant increase in the cell population in early and late apoptosis, studies that were carried out in the cell lines HeLa, HepG2, AGS, SNU-1, SNU-169 [44], PC-3 [51,52], MCF-7 [51] using concentrations of 3–50 μM and 24 h of exposure; and in another report reports the significant increase in the cell population in early, late apoptosis, and necrosis in A549 cell lines [50] using concentrations below 3 μM and 24 h of exposure. In most studies, significant induction of apoptosis was associated, through upregulation of caspase 3, 7, and 9 expressions, as well as Bax, and downregulation of Bcl-2, loss of mitochondrial membrane potential, cytochrome c release, and PARP cleavage [50,51]. Considering the above, it would be worthwhile to carry out other tests monitoring for a shorter time at different concentrations of CO.

Finally, a molecular docking study was carried out to obtain the interactions of CO and four potential therapeutic targets in cancer (Table 7, Figure 9 and Figure 10). CO obtained a higher binding energy with the HMG-CoA reductase enzyme of −7.85 kcal/mol. Meanwhile, the HMG-CoA substrate obtained an affinity of −12.54 kcal/mol and only shared the residue of Met 657 with CO. However, considering the catalytic portion of HMG-CoA reductase ranging from residues 426–888 [56], CO was bound within the catalytic portion. Therefore, it is suggested that it could act as a possible inhibitor of the enzyme that regulates the mevalonate pathway. The theoretical results obtained could suggest that CO could decrease the intermediates synthesized through the mevalonate pathway, whose function is the prenylation of the GTPase, Ras and Rho proteins [27]. On the other hand, inhibition of cholesterol synthesis could reduce tumor androgen signaling and decrease lipid rafts in cells and, to a greater extent, in tumors due to their high proliferation rate [27,28,29]. Currently, studies have been proposed supporting HMG-coA reductase as a target in cancer prevention and treatment [27,30].

For molecular docking using Bcl-2 as a target, CO obtained a favorable energy of −7.47 kcal/mol, while the inhibitor Navitoclax obtained a much more favorable affinity of −12.54 kcal/mol. However, it is suggested that CO might inhibit Bcl-2 activity, as CO and navitoclax share various residues through nonpolar interactions. The first four amino acid residues of CO are shared by non-polar-type interactions and form the hydrophobic binding groove of Bcl-2 [57]. For Mcl-1, CO obtained a binding energy of −6.77 kcal/mol, while inhibitor 9EA obtained a more favored binding energy of −10.77 kcal/mol. Analyzing the interactions of 9EA, seven amino acid residues shared by polar interactions with CO were obtained, these residues are located in the binding pocket for Mcl-1 [58], so it is inferred that CO could alter its structural conformation and inhibit its activity. Theoretical results on the interaction of CO and proteins of the BCL-2 family suggest that CO could be an antilymphoma agent by binding to amino acid residues of the binding site, inducing a different conformation and blocking the sequestration at pro-apoptotic proteins such as Bax and Bak. It is well known that Bcl-2 and Mcl-1 are proteins that are overexpressed in lymphomas [22,23,24]. Studies have pointed toward the development of more selective protein inhibitors in the BCL-2 family, including Bcl-2 and Mcl-1, to counteract the progression of lymphomas in patients [21,31,59]. Nevertheless, explorations of more specific targeted approaches for both therapeutic targets are still lacking.

Finally, molecular docking was performed for VEGFR-2, and the binding energy of the inhibitor axitinib was more favorable compared to CO, obtaining −8.57 kcal/mol and −5.8 kcal/mol, respectively. The interactions of importance for VEGFR-2 activity shared by axitinib and CO were Ala 866 and Lys 868 in the hydrophobic region within the frontal pouch [60]. Also, the interactions Asp 1046 and Cys 1045 showed possible inhibitory activity of CO on VEGFR-2. The residue Asp 1046 forms part of the DFG motif, which plays a crucial role in the binding of ATP; in the rear pocket, Cys 1045 confers the DFG-out conformation, and consequently, VEGFR-2 remains idle [33,60,61]. Therefore, our theoretical results suggest that CO may interfere with multiple signaling cascades involved in tumorigenesis and suggest that it could be used as a potential therapeutic candidate for cancer treatment. However, it should be noted that our molecular docking study, while providing crucial information for drug development, also has several limitations. Because it does not reflect all the complexity of the protein microenvironment, rigid protein, the precision of the algorithm and the water molecules.

The above results provide information about the antitumor potential of CO and yield some findings of its possible mode of action. Cytotoxic and antiproliferative properties of CO have been reported in numerous cancer lines [44], this is consistent with the activity of other terpenoids [18,19]. Terpenoids are known to prevent the interaction of carcinogens with DNA, suppress cell growth and proliferation, induce apoptosis, inhibit angiogenesis, arrest the cell cycle, and generate ROS [62]. Some of the reported mechanisms of action for CO on PC-3 and MCF-7 cancer cell lines are MAPK activation and inhibition of the PI3K/AKT/mTOR/S6K1 signaling pathway [51], signaling pathways that play an important role in cell proliferation, survival, and angiogenesis [63]. Proapoptotic activity in cancer cells has been associated with reduced activation of the transcription factor NF-κ B involved in several key cellular processes for tumorigenesis [64].

In addition, the chemical structure of CO provides the answer to its strong anti-cancer properties. It contains exocyclic methylene and epoxide functional groups, resulting in covalent binding to proteins and DNA bases [51]. This could suggest multitarget capability compared to Bcl-2 and Mcl-1 inhibitors that are specific. For example, venetoclax shows greater selectivity for Bcl-2 than for Mcl-1, however, the long-term response seems to indicate a continued risk of relapse in patients. Navitoclax is under investigation, as according to clinical study records it was associated with thrombocytopenia [58]. Another challenge of inhibitory agents is resistance, the increased expression of MCL-1 provides cells with sufficient pro-survival signaling to avoid apoptosis [65]. While CO improves the efficacy of classic drugs such as doxorubicin, this is due to its chemical structure [66]. Cyclic hydrocarbons can bind together in the cell membrane, resulting in increased bilayer permeability.

## 4. Materials and Methods

### 4.1. Chemicals

Caryophyllene oxide (mixture of isomers, PN: 53H3448-100MG), methotrexate, dimethyl sulfoxide (DMSO), L-glutamine, penicillin/streptomycin, and RMPI 1640 medium were purchased from Sigma-Aldrich, St. Louis, MO, USA. Petroleum ether, ethyl acetate, and hexane were of analytical grade and were purchased from JT Baker, Mexico City, Mexico. Bovine fetal serum was purchased from Gibco, New York, NY, USA.

### 4.2. Extraction and Isolation of Products

*A. macroprophyllata* was collected in Metapa de Domínguez, Chiapas, Mexico (14°50′00″ N 92°11′00″ W). The plant material was identified by M. C. Santiago Xolalpa of the IMSSM Herbarium of the Mexican Institute of Social Security (IMSS), with a copy of a receipt corresponding to number 16,248. The leaves were air-dried and subsequently pulverized (1.5 kg) and macerated at room temperature with petroleum ether (8L × 2). The macerated extract was filtered, collected and concentrated using a rotary evaporator (Büchi Labortechnik AG, Flawil, Switzerland) under vacuum at 40 °C to obtain 37.89 g of dry petroleum ether extract (PEAm, 2.52% yield). The results of the antilymphomatic activity of mPEA were obtained in our previous study [15]. The isolation of caryophyllene oxide (CO) was performed by preparative TLC (Merck 60F-254 silica gel, hexane-EtOAc, 85:15) through this technique, the sesquiterpene CO was obtained separated from the other compounds. For its purification, 975 mg were used to obtain an average of 26 mg of CO, this extraction and isolation procedure was carried out as many times as necessary and was performed according to the protocol previously reported [16].

### 4.3. Identification of Caryophyllene Oxide

A GC-MS analysis of PEAm was performed using the Agilent GC-MDS (Agilent 220 Technologies, Wilmington, DE, USA) and MassHunter Workstation software, version B.07.05. A 5977B gas chromatograph and a 7890B quadrupole selective mass detector were used. The column used was a 5% HP-5MS fused silica and phenylmethylsiloxane capillary column (30 m × 0.25 mm internal diameter × 0.25 μm film thickness). The carrier gas was helium at a constant flow of 1.0 mL. A 1.0 μL sample was injected into a 1/10 split injector at 250 °C. The temperature of the ion source was 230 °C, of the quadrupole was 150 °C, and of the transfer line was 250 °C. The mass detector was programmed for 70 eV electron impact ionization. The oven temperature was maintained at 130 °C for 2 min initially. The temperature was then raised at a rate of 5 °C/min to a level of 150 °C and maintained for a period of 10 min. The temperature then rose at a rate of 10 °C/min to a level of 285 °C, which we maintained again for 10 min. The compound caryophyllene oxide was identified using its mass spectra compared to NIST mass spectra libraries [67]. Once the results of the CG-MS analysis were known, a comparison of the retention factor with the authentic sigma sample was made by CO thin layer chromatography and was revealed with 10% H_2_SO_4_ and the retention factor was the same as the authentic sample (Sigma-Aldrich). To confirm the identification, we characterized the compound using spectroscopic methods (NMR ^1^H and ^13^C) (Table 2).

### 4.4. Cell-Based Assay

#### 4.4.1. Culture

The cell line U-937 (ATCC: CRL 1593.2, Middlesex, United Kingdom) was acquired. The cell line corresponds to a histiocytic lymphoma or diffuse monocytic NHL. Two million cells were used for its propagation and were seeded in an RPMI 1640 medium (Roswell Park Memorial Institute) supplemented with 2 mM of L-glutamine, 10% *v*/*v* of fetal bovine serum, 100 mM of 1% sodium pyruvate, and 1% of penicillin/streptomycin. The cells were kept in an atmosphere with a carbon dioxide (CO_2_) concentration of 5% at 37 °C.

#### 4.4.2. Cytotoxic Activity

In vitro cytotoxic activity was evaluated using the cell proliferation reagent WST-1, based on the cleavage of tetrazolium salts to formazan by the mitochondrial enzyme dehydrogenase [68]. For cell proliferation, the Rapid Cell Proliferation Kit II (Abcam, Cambridge, UK, Cat. No. ab65475) was used; 96-well plates were used, and 2 × 10^5^ cells per well were seeded in a final volume of 100 μL/well of culture medium. Cell incubation was 24 h for each experiment, and the same temperature (37 °C) and atmospheric conditions (5% CO_2_) were maintained. After incubation, treatments were added at four different concentrations. Cells treated with CO (5, 15, 30 and 45 μg/mL). Cells treated with MTX (75, 100, 150 and 175 μg/mL) as a positive control, cells treated with 1% DMSO were considered as solvent control to rule out cell death due to its effect, and cells kept only in RPMI 1640 medium. After 24 h, the manufacturer’s instructions were followed to assess viability by adding 10 μL of WST-1 reagent and incubating the cells under the above conditions. Finally, absorbance was measured at 440 nm using a microplate reader (Corning; Reynosa, Mexico). The experiments were conducted in triplicate and independently.

#### 4.4.3. Assessment of Apoptosis by Annexin V-FITC/PI Staining

First, 5 × 10 5 U-937 cells were exposed for 24 h to CC_50_ 24.25 μg/mL caryophyllene oxide and CC_50_ 118.87 μg/mL methotrexate. A commercial kit (Bio Vision, Waltham, MA, USA) was used to perform the assay, and the cells were labeled with an anti-annexin V-isothiocyanate (FITC) fluorescein antibody conjugate (FITC) and propidium iodide. After 24 h of incubation with CO, the procedure described by the commercial kit was performed, two washes were performed with 500 μL of PBS and subsequently resuspended in 100 μL of PBS containing 5 μL of annexin V and PI and then incubated for 5 min at room temperature in the dark. Gating strategy for the analysis of apoptosis/necrosis, U-937 cells were gated on FSC vs. SSC. The flow cytometer used was FACSCalibur (Becton DickinsonTM, Franklin Lakes, NJ, USA) equipped with filters at 530 nm (FITC, FL1) and 585 nm (PI, FL2). Fluorescence was analyzed and quantified in 20,000 cells in three independent experiments.

### 4.5. Animals

The animals used in the in vivo experiments were young adult male and female mice of the Balb/c strain (8-week-age, weight 20 ± 5 g). The animals were provided by the IMSS. Throughout the experiment, the animals were kept in controlled conditions with a 12 h light/dark cycle at 22 °C ± 2 °C. Food and water were available ad libitum. The experimentation procedures were approved by the Bioethics Committee of the Specialty Hospital of the National Medical Center “Siglo XXI”. The registration numbers corresponded to R-2020-3601-186 and R-2019-3601-004. The procedures were carried out under the guidelines of the official Mexican standard NOM 0062-ZOO-1999 titled “Technical Specifications for the Production, Care and Use of Laboratory Animals” [69].

### 4.6. Antilymphoma Activity

To assess antilymphoma activity, lymphoma was induced following the method by Calzada et al. [70]. First, seven groups of female mice and seven groups of male mice (*n* = 6) were randomly formed. Cell inoculation of 1 × 10^6^ U-937 cells was then performed intraperitoneally. After cell inoculation, the animals were monitored, and a record of their weekly weight was made. On day 29, the corresponding treatments were administered for each group. The groups formed for each sex were the healthy control (untreated), the healthy control group (vehicle, tween 80, 2% *v*/*v* in water), the negative control without treatment (U-937), and the positive control treated with MTX (0.1, 1.5, and 10 mg/kg) and the groups treated with CO (1.5, 10, and 15 mg/kg). The treatments were administered in a single daily dose orally for 9 days. Subsequently, the animals were kept under observation, making a record of weight, behavior, condition, and survival. Finally, on day 65, the left and right axillary and inguinal lymph nodes were removed and weighed. The activity of the antilymphoma was determined by comparing the total weight of the lymph nodes of each group against the control groups and the U-937 group. With these data we obtained the percentage of inhibition of lymph node growth for each of the doses used in the study. The ED_50_ was obtained by linear regression analysis of the percentage of inhibition of nodal growth against the dose administered for each treatment (Table 4).

### 4.7. Acute Oral Toxicity

The acute oral toxicity test was performed in accordance with the guidelines outlined by the Organization for Economic Cooperation and Development (OECD) Guideline 423 for the Assessment of Acute Oral Toxicity of Chemicals [53].

Six groups of female mice of the Balb/c strain were randomly formed (*n* = 3). The treatments were administered in a single dose orally using an esophageal cannula in mice with previous fasting and free access to water. The healthy control (untreated), the group that was administered only the vehicle (Tween 80, 2% in water), for MTX and CO, doses of 500 and 1000 mg/kg were administered. After the administration of the treatments, the animals were monitored for the next 4 h, registering any signs of toxicity (sleep, lethargy, convulsions, tremors, diarrhea, etc.) and/or death, up to 14 days. Subsequently, the animals were sacrificed, and a macroscopic observation of the organs (stomach, intestines, liver, spleen and kidneys) was carried out to report if there were any pathological changes. In addition, the organs were weighed, and the results were compared with the control group. Finally, the mean lethal dose (LD_50_) for each compound was determined, according to the classification of acute systemic toxicity recommended by the OECD in the following categories: category 1, very toxic ≤ 5 mg/kg; category 2, toxic > 5 and ≤ 50 mg/kg; category 3, harmful > 50 and ≤ 300 mg/kg; category 4, low risk > 300 and ≤2000 mg/kg; and category 5, safe or without a label > 2000 mg/kg.

### 4.8. In Silico Toxicology and Pharmaceutical Properties

The obtaining of pharmacokinetic, toxicological, and physicochemical properties through computer tools that measure the pharmacological nature and chemical compatibility with medicine of one or more small molecules support the possible recognition of candidate molecules in the development of antitumor agents. Some toxicological and pharmacokinetic parameters of CO, Molinspiration [71], SwissADME [72], ADMETlab [73], and Tox-prediction [74] were obtained.

### 4.9. Molecular Docking Studies of Caryophyllene Oxide

The chemical structures of the ligand caryophyllene oxide (CID: 1742210), hydroxymethylglutaril-CoA substrate (CID: 445127), 1XJ or Navitoxlax (CID: 24978538), 9EA (CID: 68938909), and AXI or Axitinib (CID: 6450551) were retrieved from the PubChem chemical library (https://pubchem.ncbi.nlm.nih.gov/) (accessed 11 May 2024).

The ligands were optimized and subjected to energetic and geometric minimization using the Avogadro software 2 [75]. Subsequently, the proteins or targets were downloaded from the Protein Data Bank (http://www.rcsb.org/) (accessed 13 May 2024). The targets used in the in silico study were hydroxymethylglutaryl-CoA reductase (RCSB, PDB ID: 1DQ9), Bcl-2 (RCSB, PDB ID: 4LVT), Mcl-1(RCSB, PDB ID: 5VKC), and VEGFR-2 (RCSB, PDB ID: 4AG8). The proteins were then prepared to maintain the conditions required in the study. To do this, the ions and total water molecules that they did not need for their catalytic activity were extracted. All polar hydrogen atoms, ionized in a basic environment (pH = 7.4), were aggregated, and Gasteiger charges were assigned. The output topologies calculated from the previous steps were used as input files for the docking simulations.

Theoretical molecular docking experiments were carried out using Autodock 4.2 software [76]. For the enzyme HMG-coA reductase, a grid box of 126 × 126 × 126 Å^3^ was used; and for Bcl-2, Mcl-1, and VEGFR-2 a grid box of 90 × 90 × 90 Å was used at each spatial coordinate, with a grid point spacing of 0.375 Å. The Lamarckian genetic algorithm was used as a scoring function with a random initial population of 100 individuals and a maximum number of energy assessments of 1 × 10^7^ cycles. The analysis of the interactions in the enzyme/inhibitor complex was visualized with the PyMOL software (The PyMOL Molecular Graphics System, Ver 2.0, Schrödinger, LLC, DeLano Scientific, San Carlos, CA, USA). Molecular docking validation was performed by recoupling the cocrystallized ligand on the receptor (HMG-CoA substrate, Navitoclax, 9EA, and axitinib). The RMSD was calculated and a reliable range within 2 Å was considered, the calculation was performed by superimposing the cocrystallized ligand with the lowest energy and it was observed if it maintained the same binding position.

### 4.10. Statistical Analysis

The results were expressed as mean ± standard error of six measurements. The results were analyzed using the GraphPad Prisma version 8.4 program (GraphPad software, San Diego, CA, USA). One-way ANOVA as well as Dunnet’s multiple comparison tests with a value of *p* ˂ 0.05 were used to establish that there were significant differences between the study groups. The CC_50_ and ED_50_ were obtained by regression analysis and the LD_50_ was calculated by linear interpolation of the percentage mortality values for each dose.

## 5. Conclusions

A comprehensive analysis of our obtained results suggests that caryophyllene oxide could be a potential therapeutic agent in the treatment of cancer. Its anticancer properties are mediated by the induction of late apoptosis and necrosis. In addition, acute oral toxicity results and computational toxicological parameters indicated that CO is a molecule that presents a wide dose range for human consumption. In addition, theoretical results suggest that caryophyllene oxide is a multitarget molecule that acts on important residues of the Bcl-2 and Mcl-1 proteins and binds to residues of the active site of HMG-CoA reductase and VEGFR-2. The results of the identification of caryophyllene oxide in *A. macroprophyllata* support its use in traditional medicine as an antitumor agent, and this terpenoid could be a potential agent for U-937 cell-associated lymphoma. However, given the limitations of the studies conducted, more evidence and data would be needed to confirm the safety and mechanism of action of sesquiterpene.

## Figures and Tables

**Table 1:** GC-MS, retention times, and percentage in area of caryophyllene oxide identified from petroleum ether extract from *A. macroprophyllata* leaves.

Compound Name	R.T * (min)	Area %	Molecular Weight	Molecular Formula
Caryophyllene oxide	7.93	2.03	220.35	C_15_H_24_O

* R.T: Retention time.

**Table 2:** ^1^H and ^13^C (300 MHz) NMR data of caryophyllene oxide in CDCl_3_.

Position	Caryophyllene Oxide	
	δ_H_, mult. (*J* in Hz)	δ_C_, Type
1	1.78 (H, m, 5.31)	50.86 (CH)
2	1.71 (2H, m, 1.05)	26.24 (CH2)
3	1.6 (2H, m, 1.69)	39.14 (CH2)
4		60 (C)
5	2.86 (H, m)	63.4 (CH)
6	2.08 (2H, m, 3.2)	29.6 (CH2)
7	2.34 (2H, m, 1.01)	30.8 (CH2)
8		151.7 (C)
9	2.62 (H, m, 1.04)	48.58 (CH)
10	2.04 (2H, m, 1.05)	39.33 (CH2)
11		34.52 (C)
12	4.93 (3H, m, 0.96)	17.75 (CH3)
13	1.21 (2H, s)	112.07 (CH2)
14	0.99 (3H, s)	26.0 (CH3)
15	0.97 (3H, s)	26.1 (CH3)

**Table 3:** Median cytotoxic concentration calculated after 24 h of exposure against U-937 cells from caryophyllene oxide (CO) and methotrexate (MTX).

Sample	CC_50_ (µg/mL) ^a^
CO	24.25 ± 0.37 *
MTX	118.87 ± 0.26

^a^ CC_50_: Median cytotoxic concentration causing 50% cell death. Calculated by linear regression analysis of percentage mortality against concentration. Data are expressed as mean ± SEM, (*n* = 3). * *p* < 0.05 vs. MTX.

**Table 4:** Results of the antilymphoma activity after administration of MTX y CO obtained from the leaves of *A. macroprophyllata* on male and female mice inoculated with U-937 cells.

Treatment	ED_50_ (mg/kg) ^a^	
	Female	Male
CO	6.14 ± 0.52 *	5.46 ± 0.42 *
MTX	1.24 ± 0.038	0.94 ± 0.023

^a^ ED_50_: Median effective dose (causing 50% of population to have the desired pharmacological effect). Linear regression analysis of percentage of inhibition of lymph node growth against the dose administered for each treatment. The results are presented as mean ± SEM, (*n* = 6). * *p* < 0.05 vs. MTX.

**Table 5:** Acute oral toxicity from MTX and CO obtained from the leaves of *A. macroprophyllata*.

Sample	LD_50_ (mg/kg) ^a^	TI ^b^ Female	TI ^b^ Male
CO	>1000	162.86	183.15
MTX	335.04 ± 0.39	270.19	356.42

^a^ LD_50_: Median lethal dose (causing 50% animal death). Calculated by linear regression analysis of percentage mortality against the dose administered for each treatment. The results are presented as mean ± SEM, (*n* = 3). ^b^ TI: therapeutic index calculated as LD_50_ (acute oral toxicity)/ED_50_ (antilymphoma activity).

**Table 6:** Physicohemical, pharmacokinetic, and toxicologic predictive values of caryophyllene oxide ^a^.

Physicochemical		Pharmacokinetic	
TPSA	12.53	Human intestinal absorption	High
Lipophilicity (logP)	4.53	BBBp	Yes
Water solubility (logS)	−5.29	Volume of distribution	1.27
Rotatable bonds	0	Plasma protein binding	82.26%
Number of H donors	0	CYP1A2 inhibitor	No
Number of H-bond acceptors	1	CYP2C19 inhibitor	Yes
Druglikeness		CYP2C19 substrate	Yes
Lipinski	Yes; 0 violation	CYP2C9 inhibitor	Yes
Ghose	Yes	CYP2D6 inhibitor	No
Veber	Yes	CYP2D6 substrate	Yes
Egan	Yes	CYP3A4 inhibitor	No
Toxicity		Clearance	6.45
Mutagenic	No	T1/2	0.17
Carcinogenic	No		
Rat Oral Acute Toxicity	No		
H-HT	No		
Predicted Toxicity Class ^b^	5		

Topological polar surface area (TPSA), logarithm of the n-octanol/water distribution coefficient (logP), blood–brain barrier permeability (BBBp), human hepatotoxicity (H-HT). ^a^ Predictions were based on Molinspiration, SwissADME, ADMETlab and Tox-prediction web servers, and they were ^b^ Class I: fatal if swallowed (LD_50_ ≤ 5), Class II: fatal if swallowed (5 < LD_50_ ≤ 50), Class III: toxic if swallowed (50 < LD_50_ ≤ 300), Class IV: harmful if swallowed (300 < LD_50_ ≤ 2000), Class V: may be harmful if ingested (2000 < LD_50_ ≤ 5000), Class VI: non-toxic (LD_50_ > 5000).

**Table 7:** Interactions of caryophyllene oxide with amino acid residues on the binding sites of Bcl-2, Mcl-1, and HMG-coA reductase and VEGFR-2.

Compound	HMG-coA reductase			
	ΔG (kcal/mol)	H-BR	NPI	RMSD
CO	−7.85	Glu 559, Asn 658, Gly 765, Gln 766, Asp 767, Gln 770, Ile 802, Gly 803, Val 805, Gly 806, Gly 807, Gly 808	Ala 654, Met 657	-
HMG-CoA- substrate	−9.21	Pro 477, Tyr 479, Glu 528, Asn 529, Cys 561, Leu 562, Ser 565, Asn 567, Arg 590, Met 657, Ser 684, Asn 686, Asp 690, Lys 691, Lys 692, Val 720, Lys 735, Ala 751, His 752, Asn 755, Ser 852, Ala 856, Leu 857, His 861	Ala 564, Arg 568, Arg 571, Lys 722, Leu 853, Leu 862	1.10
Compound	Bcl-2			
	ΔG (kcal/mol)	H-BR	NPI	RMSD
CO	−7.47	Asp 108, Phe 147, Glu 149	Phe 101, Phe 109, Met 112, Val 130, Leu 134, Ala 146, Phe 150, Val 153	-
Navitoclax (ABT-263)	−12.54	Asp 100, Arg 104, Tyr 105, Asp 108, Phe 109, Glu 133, Asn 140, Trp 141, Gly 142, Val 145, Phe 195, Leu 198, Tyr 199	Ala 97, Phe 101, Met 112, Val 130, Leu 134, Arg 143, Ala 146, Phe 150	1.20
Compound	Mcl-1			
	ΔG (kcal/mol)	H-BR	NPI	RMSD
CO	−6.77	Leu 246, Thr 266, Gly 271, Leu 290, Ile 294	Phe 228, Met 231, Leu 235, Val 249, Met 250, Val 253, Leu 267, Phe 270, Val 274	-
9EA	−10.77	Val 216, Phe 228, Leu 235, Val 249, Phe 254, Gly 262, Arg 263, Val 265, Gly 271, Leu 290, Ile 294, Phe 318, Phe 319	Val 220, His 224, Ala 227, Met 231, Leu 246, Met 250, Val 253, Thr 266, Leu 267, Phe 270, Val 274	1.45
Compound	VEGFR-2			
	ΔG (kcal/mol)	H-BR	NPI	RMSD
CO	−5.8	Glu 885, Leu 1035, Asp 1046	Val 848, Ala 866, Lys 868, Leu 882, Leu 889, Val 899, Val 914, Val 916, Cys 1045, Phe 1047	-
Axitinib (AG-013736)	−8.57	Glu 885, Leu 889, Glu 917, Phe 918, Gly 922, Asn 923, Asp 1046	Leu 840, Val 848, Ala 866, Lys 868, Val 899, Val 914, Val 916, Cys 919, Leu 1035, Cys 1045, Phe 1047	1.8

ΔG: Binding energy (kcal/mol); H-BR: H-binding residues; NPI: Nonpolar interactions; Ala: Alanine; Asp: Aspartate; Asn: Asparagine; Arg: Arginine; Cys: Cysteine; Gln: Glutamine; Lys: Lysine; Thr: Threonine; Ser: Serine; Trp: Tryptophan; Leu: Leucine; His: Histidine; Gly: Glycine; Glu: Glutamic acid; Ile: Isoleucine; Tyr: Tyrosine; Phe: Phenylalanine. All results were obtained through docking approaches.

## Data Availability

The data presented or additional data in this study are available on request from the corresponding author.
